# A Retrospective Analysis of Blood Component Utilization and Transfusion-Related Factors in a Diverse Patient Population

**DOI:** 10.3390/healthcare14010125

**Published:** 2026-01-04

**Authors:** Yazeed Abdulaziz Almujali, Nancy Mohamed Darwish, Abdulqader Ali Ba Abbad, Hani Tamim, Muhammad Raihan Sajid, Rimah Abdullah Saleem, Abdulwahab Binjomah, Kamel Aldosari

**Affiliations:** 1 College of Medicine, Alfaisal University, Riyadh 11533, Saudi Arabia; 2 Department of Internal Medicine, Clinical Research Institute, American University of Beirut Medical Center, Beirut P.O. Box 11-0236, Lebanon; 3 Bacterial Diseases and Special Pathogens Department, Public Health Authority, Riyadh 13351, Saudi Arabia

**Keywords:** blood transfusion, demographics, clinical parameters, age, platelets, hemoglobin, packed red blood cells, fresh frozen plasma

## Abstract

Background/Objectives: This retrospective observational study aimed to analyze blood component utilization patterns and investigate associations between patient demographics, clinical parameters, and transfusion requirements at a major tertiary care center. Methods: The study analyzed data from 2867 patients at King Saud Medical City, Riyadh. Utilization patterns and correlations were assessed for packed red blood cells (PRBCs), fresh frozen plasma (FFP), platelets, and cryoprecipitate. Results: Significant correlations were found between age, hemoglobin levels, platelet counts, ferritin levels, and transfusion of specific components. Elderly patients received more PRBCs, FFP, and platelets, while younger patients received more cryoprecipitate. Hemoglobin < 7 g/dL was the most common trigger for PRBC transfusion. Platelet transfusions correlated strongly with thrombocytopenia, and elevated ferritin levels correlated with higher platelet transfusion volumes. Patients with hematologic malignancies, β-thalassemia, and sickle cell disease demonstrated distinct transfusion profiles. Conclusions: These findings underscore the importance of tailored, evidence-based transfusion strategies to optimize blood use and minimize risk.

## 1. Introduction

Blood products, including packed red blood cells (PRBCs), fresh frozen plasma (FFP), platelets, and cryoprecipitate, are essential in modern medicine, significantly reducing morbidity and mortality in various clinical settings. Blood transfusion plays a role in treating acute bleeding, blood loss during surgery, and blood disorders like severe anemia, leukemia, and hemophilia. It is also important for treating patients receiving chemotherapy, organ transplants, and intensive care [1]. Despite the therapeutic benefits of blood transfusion, there are possible risks that can be observed, such as immune reactions, infections, and transfusion-related acute lung injury (TRALI), which require strict regulations to ensure safety and effectiveness [2].

Blood transfusions play a key role in the maintenance of hemodynamic stability and the management of various medical conditions. PRBCs, for example, are vital in improving low oxygen in tissues due to unexpected blood loss or long-term anemia due to trauma and surgery cases. However, excessive use of PRBCs could lead to serious complications, including infections. This illustrates the importance of correct usage based on proven transfusion criteria [3]. In contrast, FFP, is essential to treat bleeding diathesis, massive transfusions, and liver disease. However, it should be used carefully to prevent edema and allergic responses [4].

In addition, platelets and cryoprecipitate are utilized in blood transfusion. For instance, platelets are mainly used to treat low platelet counts, while cryoprecipitate helps patients with low fibrinogen levels [5]. These products have shown a positive impact on survival rates, especially in patients with blood clotting disorders. Yet, excessive transfusion of these products could lead to fever or deadly complications like TRALI [6].

Transfusion practices worldwide are based on extensive research and established protocols [7]. However, comprehensive data on hospital-wide blood utilization patterns in Saudi Arabia remain limited. While previous studies have focused on specific patient groups (e.g., hemoglobinopathies) or smaller scales, this study provides a large-scale, institution-wide analysis of transfusion practices at a major tertiary referral center, capturing a broader and more diverse patient population. By identifying knowledge gaps in local transfusion therapy, we can gain insights into enhancing patient safety, resource allocation, clinical outcomes, and development of institution-specific protocols that address the unique demographics and disease burden of the region.

Therefore, this study aims to analyze blood component utilization patterns and their associations with demographic and clinical parameters at King Saud Medical City, to inform local transfusion guidelines and optimize patient care.

## 2. Methods

### 2.1. Study Design

This study adopted a retrospective observational design to investigate blood transfusion practices in a diverse patient population at KSMC. King Saud Medical City (KSMC) is a large public tertiary care and trauma referral center in Riyadh, serving as a national referral center for complex cases including major trauma, orthopedic surgery, hemoglobinopathies, and critical care. The data was collected between January 2022 and December 2022. The study considered several parameters such as demographics, laboratory parameters and diseases that require blood transfusion.

### 2.2. Data Collection

Data was collected from the regional laboratory, blood bank, and medical records department at KSMC. Several parameters have been included in this study, such as age, gender, nationality, length of stay, admit date, and discharge date. Number of blood transfusions, blood components, and laboratory blood exams were also collected through the health information system (Medisys, New York, NY, USA) including (Patients’ ABO Blood Group/RH, PRBC units transfused, Platelets units transfused (single-donor apheresis or pooled derived from whole blood), FFP units transfused, Cryoprecipitate units transfused, Hemoglobin for patients, Platelets counts for patients, Ferritin for patients).

All patients who received blood transfusions during the study period were included in this study, and patients with incomplete or missing data and those who did not receive any blood transfusions were excluded from this study. Laboratory values such as ferritin and platelet counts were not universally available for all transfused patients. Missing data were assumed to be missing at random, as their absence was primarily due to the lack of clinical indication for the test rather than patient characteristics related to the outcome. However, the potential for selection bias cannot be ruled out, and correlation analyses should be interpreted in light of the available sample for each parameter.

During the study period, KSMC transfusion protocols were aligned with international standards, primarily following AABB guidelines for PRBC (trigger Hb < 7 g/dL for stable inpatients, or <8 g/dL in critically ill or cardiac patients) and platelet transfusion (trigger <10–20 × 10^3^/μL for prophylaxis in non-bleeding patients, or <50 × 10^3^/μL for active bleeding or invasive procedures).

### 2.3. Study Variables

The primary dependent variables in this study included the type and number of blood components transfused, i.e., packed red blood cells (PRBC), platelets, fresh frozen plasma (FFP), and cryoprecipitate (CRYO). Independent Variables included age, gender, nationality, blood type (ABO RH), and clinical outcomes. To study age-specific transfusion practices, age was first divided into six groups. Gender was categorized as either male or female in the second place. Third, the patient’s nationality was determined by their country of origin. Fourth, blood type was categorized using the Rh factor and ABO blood type. Ultimately, the clinical outcomes were divided into five categories: death, discharge or transfer to a residential aging service, discharge or transfer to other health care accommodation, left against medical advice, and treatment. Covariates included hemoglobin levels, platelet counts, ferritin levels, and length of hospital stay.

Transfusion triggers were inferred from laboratory values proximate to the transfusion order. A ‘trigger’ for PRBC transfusion was operationally defined as a hemoglobin level < 7 g/dL measured within 24 h prior to transfusion, in accordance with common institutional guidelines. However, the retrospective design did not allow for capture of clinical symptoms or active bleeding that may have contributed to the decision, which is a limitation of this operational definition.

### 2.4. Data Analysis

Data analysis was conducted using IBM SPSS version 26.0. Descriptive statistics, including means, medians, standard deviations, and interquartile range are statistical measures used to describe and analyze data. The Mann–Whitney U test was used to compare transfusion volumes between genders. The Kruskal–Wallis test was used to compare transfusion volumes across age groups and diagnostic categories.

This study was conducted following ethical standards and principles. The research protocol was approved by the hospital’s ethics committee (Reference number H1RE-29-Dec22-01) in Jan 2023, at KMSC. Patient data were anonymized to ensure confidentiality and comply with data protection regulations.

## 3. Results

The mean age of the patients was 33 years, where both genders and nationality represented the study population almost equally (female 53% and male 47%) (Saudi, non-Saudi) (Table 1).

In addition, Table 2 provides a detailed overview of the blood products transfused, key laboratory parameters, and the length of stay for the 2867 patients included in this study.

The table outlines the mean and standard deviation (SD) for the quantity of blood products received by the patients. On average, patients received 3 units of packed red blood cells (PRBC) with an SD of 3. The median number of PRBC units, represented by IQR (interquartile range), was 2 units, with a range of 1–3 units. Patients also received an average of 3 units of platelets, with an SD of 4 units. The median number of platelet units was 2, with an IQR of 1–4 units. Additionally, patients received 7 units of fresh frozen plasma (FFP) on average, with an SD of 7 units, and the median number of FFP units was 4, with an IQR of 2–8 units. Cryoprecipitate (CRYO) was also administered to patients, with an average of 10 units and an SD of 7 units. The median number of CRYO units was 9, with an IQR of 6–12 units.

### 3.1. Laboratory Parameters

The table provides information on several key laboratory parameters. Patients had an average hemoglobin level of 7 g/dL, with an SD of 2 g/dL. The median hemoglobin level was 7 g/dL, with an IQR of 6–8 g/dL. Platelet counts were also reported, with an average of 58 × 10^3^/mm^3^ and an SD of 88 × 10^3^/mm^3^. The median platelet count was 24 × 10^3^/mm^3^, with an IQR of 11–57 × 10^3^/mm^3^. Additionally, ferritin levels were measured, with an average of 961 ng/mL and an SD of 940 ng/mL. The median ferritin level was 698 ng/mL, with an IQR of 38–2000 ng/mL.

The distribution of primary diagnostic categories is presented in Table 3. Hemoglobinopathies collectively represented the largest group (30.0%), followed by major surgical procedures, critical care admissions, and other medical conditions.

Table 4 presents the results of Pearson correlation analyses that explore the relationships between received blood units (PRBC, Platelets, FFP, and CRYO) and several key variables, including age, hemoglobin levels, platelet count, ferritin levels, and the length of stay. The table provides correlation coefficients, significance levels, and the number of cases (N) for each analysis. Varying sample sizes (N) in Table 4 are due to missing laboratory data for some patients who received the specified blood component. Correlations were calculated using Spearman’s rho.

### 3.2. Age

The analysis reveals statistically significant correlations between age and the transfusion of various blood components. Transfusion volumes differed significantly across age groups (Kruskal–Wallis, *p* < 0.01). Age positively correlated with PRBC units (r = 0.156, *p* < 0.0001) and negatively correlated with Platelets (*p* = 0.038). Additionally, age shows a positive correlation with FFP units (*p* = 0.026) and a stronger negative correlation with CRYO units (r = −0.271, *p* = 0.032).

### 3.3. Hemoglobin (Hb) Levels

Hemoglobin levels demonstrate significant correlations with the transfusion of different blood components. Hemoglobin is negatively correlated with PRBC units (r = −0.266, *p* = 0.000) and Platelets (r = −0.115, *p* = 0.041). Hemoglobin also exhibits a negative correlation with FFP units (r = −0.211, *p* = 0.000) but does not significantly correlate with CRYO units (r = −0.077, *p* = 0.550).

### 3.4. Platelet Count

Platelet count shows statistically significant correlations with the transfusion of Platelets (r = −0.241, *p* = 0.000) and FFP (r = −0.193, *p* = 0.008). However, it does not significantly correlate with PRBC or CRYO units (r = −0.11, *p* = 0.053 and r = −0.241, *p* = 0.096, respectively).

### 3.5. Ferritin Levels

Ferritin levels exhibit a strong positive correlation with the transfusion of Platelets (r = 0.423, *p* = 0.000). However, ferritin levels do not significantly correlate with the transfusion of PRBC, FFP, or CRYO units (r = 0.016, *p* = 0.632; r = −0.053, *p* = 0.634; r = 0.103, *p* = 0.764, respectively).

### 3.6. Length of Stay

The Table 4 also presents data on the length of stay for the patients. On average, patients stayed for 12 days, with an SD of 20 days. The median length of stay was 5 days, with an IQR of 1–15 days. The length of stay in days is significantly correlated with all blood components. It shows a positive correlation with PRBC units (r = 0.496, *p* = 0.000) and Platelets (r = 0.310, *p* = 0.000). However, it does not significantly correlate with FFP or CRYO units (r = 0.067, *p* = 0.166; r = 0.076, *p* = 0.554, respectively).

Table 4 outlines Pearson correlation analyses for 2867 patients, investigating relationships between received blood units and key variables:

Age: Positively correlated with PRBC units, FFP units; negatively correlated with Platelets and CRYO units.

Hemoglobin (Hb) Levels: Negatively correlated with PRBC units, Platelets, FFP units; no significant correlation with CRYO units.

Platelet Count: Negatively correlated with Platelets, FFP units; no significant correlation with PRBC or CRYO units: Strong positive correlation with Platelets; no significant correlation with PRBC, FFP, or CRYO units.

Length of Stay: Positively correlated with PRBC units, Platelets; no significant correlation with FFP or CRYO units.

Given that elevated ferritin can reflect acute inflammation, iron overload, or malignancy, the correlation with platelet transfusion was explored in three clinical subgroups based on primary diagnosis: (1) patients with acute conditions (e.g., trauma, ICU admission), (2) patients with hematologic malignancies or bone marrow failure, and (3) patients with hemoglobinopathies (β-thalassemia, sickle cell disease) on chronic transfusion regimens. This post hoc analysis suggested that the positive correlation was strongest in subgroup (2) (patients with malignancies) and present in subgroup (1) (acute illness) but absent in subgroup (3) (chronic hemoglobinopathies). These observations support the hypothesis that the ferritin–platelet association is driven by acute inflammatory states or disease burden rather than chronic iron overload.

## 4. Discussion

Given the observational nature of this study, all reported relationships should be interpreted as associations, not causative effects. Our study highlights significant correlations between patient characteristics and blood component utilization in a diverse Saudi population. The positive correlation between age and transfusion of PRBCs, fresh frozen plasma (FFP), and platelets aligns with previous reports of higher transfusion needs in older patients, likely attributable to a higher burden of comorbid conditions and surgical interventions [2]. Conversely, the negative correlation between age and cryoprecipitate (CRYO) use may reflect its specific clinical indications, which are more common in younger trauma or obstetric patients [8].

The observed association between older age and increased PRBC, FFP, and platelet use is consistent with the epidemiology of aging, where a higher prevalence of chronic illnesses and major surgical procedures frequently necessitates blood support [4,7,9]. Our findings are corroborated by a local study in which patients aged 50 and older accounted for 41.3% of all transfusions, emphasizing the need for age-conscious transfusion protocols [10]. Furthermore, the inverse relationship between hemoglobin (Hb) levels and PRBC transfusions reinforces established clinical guidelines that recommend transfusion for significant anemia [7,11]. In our cohort, a hemoglobin level < 7 g/dL was the most common proximate laboratory trigger for PRBC transfusion, consistent with both local practice and international recommendations [11,12]. It is important to note, however, that clinical decisions also incorporate patient symptoms and overall condition, factors not fully captured in this analysis.

Hereditary hemoglobinopathies constituted the largest diagnostic group requiring transfusion support. The high utilization of PRBCs in patients with β-thalassemia and sickle cell disease predominantly reflects scheduled chronic transfusion programs aimed at preventing complications such as iron overload, stroke, and acute chest syndromes [13,14,15]. A smaller proportion of transfusions in this group were for acute events or perioperative support. This pattern underscores the central role of transfusion medicine in the lifelong management of these disorders and aligns with prior studies highlighting the substantial and ongoing blood product demands of this patient population [13,14]. The management of transfusional iron overload in this cohort, guided by ferritin levels and imaging, remains a critical aspect of their care [16]. Platelet count was strongly and inversely correlated with platelet transfusion volume, confirming standard clinical practice where transfusions are administered to mitigate the risk of bleeding in thrombocytopenic patients [9]. This is supported by studies in critically ill populations, where a high prevalence of thrombocytopenia drives platelet utilization [17,18,19].

The strong positive correlation between pre-transfusion ferritin levels and platelet transfusion volume is a notable finding. Ferritin is a nonspecific acute-phase reactant elevated in conditions such as inflammation, infection, malignancy, and iron overload [17,18,20]. To contextualize this association, a post hoc subgroup analysis was performed. This analysis suggested the correlation was most pronounced in patients with acute conditions (e.g., trauma, sepsis) and those with hematologic malignancies, where elevated ferritin likely serves as a marker of systemic inflammation or high disease burden prompting platelet support [20,21,22,23]. In contrast, the association was absent in patients with hemoglobinopathies on chronic transfusion regimens, for whom ferritin primarily reflects transfusional iron overload. This distinction is critical: in the chronic setting, ferritin guides iron chelation therapy [16,20], while in acute or oncologic settings, it may indirectly signal an inflammatory state associated with consumptive or suppressive thrombocytopenia [17,23]. Therefore, this association likely reflects the underlying inflammatory milieu or disease severity in patients requiring platelet support, rather than indicating a direct causal mechanism. Further prospective research is needed to disentangle these contributing factors.

Longer hospital stays were positively correlated with increased transfusion of PRBCs and platelets. This association underscores that transfusion requirements are closely linked to care complexity, as prolonged admissions often involve major surgeries, chemotherapy, or management of chronic critical illnesses that necessitate blood product support [22,24,25]. This relationship is bidirectional, as the need for transfusion can also be a marker of illness severity that itself prolongs hospitalization [26,27]. In trauma and massive transfusion scenarios, specific blood component ratios are employed, which can influence overall utilization and outcomes [22,24].

It is important to acknowledge the limitations of our study, which impact the generalizability of the findings. This research was conducted at a single, large tertiary care center that serves as a national referral center for hemoglobinopathies and complex trauma. Consequently, the observed transfusion patterns—particularly the high prevalence of transfusion-dependent hemoglobinopathies—may not be directly applicable to community hospitals or populations with a different clinical case mix.

In conclusion, our findings align with and extend the existing literature on transfusion practices. It is well-established that age, hemoglobin levels, and platelet counts significantly influence transfusion decisions [8,28,29,30]. The novel association between ferritin and platelet transfusion volume adds a layer of complexity, suggesting ferritin may be a useful indirect marker of the inflammatory states that often necessitate platelet support. These results highlight the multifactorial nature of transfusion decisions and reinforce the importance of tailored, evidence-based strategies to optimize blood use and patient outcomes.

## 5. Conclusions

This retrospective analysis identifies significant associations between patient demographics, clinical parameters, and blood component utilization in a tertiary care setting. Key findings include the correlation of older age with increased PRBC, FFP, and platelet use; the inverse relationship between hemoglobin levels and PRBC transfusion; and a novel association between elevated ferritin and platelet transfusion volume, possibly indicative of underlying inflammation or iron overload. While highlighting the multifactorial nature of transfusion decisions, the study is limited by its single-center, observational design and the lack of multivariate adjustment. These findings underscore the need for tailored, evidence-based transfusion strategies and suggest avenues for future prospective, multi-center research to establish guidelines and control for confounding factors.

### Limitations

Several limitations to this study should be considered. First, due to the retrospective nature of the research, the analysis was dependent on data previously recorded in electronic health records. This reliance introduces the possibility of inaccuracies or incomplete documentation, which may have affected the reliability of certain variables. Second, the study was conducted at a single institution, which may limit the generalizability of the findings to other clinical settings or broader patient populations. Future research involving multiple centers and prospective data collection would be valuable in validating and extending the applicability of these results. A key limitation is the absence of multivariable regression analysis, which precludes controlling for potential confounding among interrelated variables such as age, diagnosis, and laboratory parameters. Future studies should employ such models to identify independent predictors of transfusion requirements.

## Figures and Tables

**Table 1:** Characteristics of the patients (n = 2867).

Parameter		Frequency (%)
Age, y	0–1	188 (6.6%)
	2–12	292 (10.2%)
	13–17	266 (9.3%)
	18–40	1282 (44.7%)
	40–65	560 (19.5%)
	65–106	279 (9.7%)
Age (Mean ± SD)		33 ± 21
Gender	Female	1520 (53%)
	Male	1347 (47%)
Nationality	Saudi Arabia	1378 (48.1%)
	Non Saudi	1297 (45.2%)
	Unknown	192 (6.7%)

**Table 2:** Blood Product Utilization and Clinical Parameters (n = 2867).

Parameter	Mean ± SD
PRBC	3 ± 3
Platelets	3 ± 4
FFP	7 ± 7
CRYO	10 ± 7
Hemoglobin, (g/dL)	7 ± 2
Platelets, 10^3^/mm^3^	58 ± 88
Ferritin, ng/mL	961 ± 940
Length of stay (days)	12 ± 20

**Table 3:** Primary Diagnostic Categories among Patients Receiving Blood Products (n = 2867).

Diagnostic Category	Frequency	Percentage (%)	Representative Sub-Diagnoses
Hereditary Hemoglobinopathies	859	30.0%	Beta thalassemia, sickle cell disease
Surgical & Procedure-Related	423	14.8%	Caesarean section, traumatic fracture repair, GI surgery
Critical Medical Conditions	363	12.7%	Sepsis, acute MI, pneumonia, GI hemorrhage, renal failure
Non-Hemoglobinopathy Anemias	301	10.5%	Unspecified anemia, iron deficiency anemia, aplastic anemia
Obstetric & Peripartum Conditions	261	9.1%	Delivery-related anemia, placenta previa, abortion
Trauma & Acute Injury	213	7.4%	Traumatic fracture, burn injury, traumatic hemorrhage
Hematologic Malignancies	155	5.4%	Acute leukemia, lymphoma, malignant neoplasms
Other Medical Diagnoses	292	10.2%	Diabetes complications, infections, inflammatory disorders, benign neoplasms

**Table 4:** Correlations between Received Blood Units with Age, Laboratory Data, and Length of Stay.

Correlations					
Parameter		PRBC Transfused	Platelets Transfused	FFP Transfused	CRYO Transfused
Age	Pearson Correlation	0.156 **	−0.116 *	0.107 *	−0.271 *
	Sig. (2-tailed)	0.000	0.038	0.026	0.032
	N	2867	317	431	63
Hemoglobin, g/dL	Pearson Correlation	−0.266 **	−0.115 *	−0.211 **	−0.077
	Sig. (2-tailed)	0.0001	0.041	0.0001	0.550
	N	2523	316	431	63
Platelets, ×10^3^/mm^3^	Pearson Correlation	−0.11	−0.241 **	−0.193 **	−0.241
	Sig. (2-tailed)	0.053	0.000	0.008	0.096
	N	309	309	187	49
Ferritin, ng/mL	Pearson Correlation	0.016	0.423 **	−0.053	0.103
	Sig. (2-tailed)	0.632	0.000	0.634	0.764
	N	850	75	83	11
Length of stay, days	Pearson Correlation	0.496 **	0.310 **	0.067	0.076
	Sig. (2-tailed)	0.000	0.000	0.166	0.554
	N	2867	317	431	63

**. Correlation is significant at the 0.01 level (2-tailed). *. Correlation is significant at the 0.05 level (2-tailed).

## Data Availability

The original contributions presented in this study are included in the article. Further inquiries can be directed to the corresponding author.
